# Differential response of patient-derived primary glioblastoma cells to metabolic and adhesion inhibitors

**DOI:** 10.1007/s10238-025-01736-6

**Published:** 2025-06-26

**Authors:** Rasha Rezk, Fikret Basar, John Mediavillo, Rebecca Donaldson, Colin Watts, Kristian Franze, Alexandre J. Kabla

**Affiliations:** 1 Department of Biology, York Biomedical Research Institute, University of York, York, UK; 2 Department of Engineering, University of Cambridge, Cambridge, UK; 3 Birmingham Brain Cancer Program, Institute of Cancer and Genomic Sciences, University of Birmingham, Birmingham, UK; 4 Department of Physiology, Development and Neuroscience, University of Cambridge, Cambridge, UK

**Keywords:** Glioblastoma, Mechanobiology, Drug discovery

## Abstract

This study aims to investigate the cellular response of Glioblastoma (GBM) to adhesion and metabolic inhibitors, focusing on cell migration and matrix adhesion properties. GBM is the most common incurable brain tumor. Despite decades of research into GBM’s chemical and molecular classification, identifying mechanisms of drug resistance has been challenging. Studies on inhibitors targeting cancer cell migration and proliferation rarely consider the heterogeneous migration properties among cells, which may impact patient responses to treatment. In this work, tissue samples were obtained from spatially distinct locations with different 5-aminolevulinic acid (5-ALA) fluorescent intensities—including strongly fluorescent tumor cores, a weakly fluorescent tumor rim, and non-fluorescent tumor margins. These samples were previously shown to be associated with significantly different motility and adhesion properties. We tested the response of tumor cells to adhesion and metabolic inhibitors using metabolic MTT and Cell Titer Glo viability assays, respectively. We also monitored cell survival using time-lapse microscopy, while culturing them on low-modulus polydimethylsiloxane (representing the stiffness of brain tissue). Metabolic viability assays revealed substantial heterogeneity in drug potency across cells from different regions of the tumor. Highly fluorescent tumor core cells were significantly more resistant to an F0F1 ATP synthase inhibitor (Gboxin), and a FAK inhibitor (GSK2256098), while their proliferation ceased post-treatment in vitro. In contrast, cells derived from non-fluorescent tumor margins exhibited higher potency for the ATP synthase inhibitor (Gboxin), but their proliferation persisted post-treatment. Our study demonstrates a correlation between the adhesive and migration properties of cells and their sensitivity to therapeutics in different regions of the tumor within individual patients and between patients with GBM.

**Supplementary Information:**

The online version contains supplementary material available at 10.1007/s10238-025-01736-6.

## Introduction

Glioblastoma (GBM) is comprised of genetically [1–3] and biomechanically [4] heterogeneous cell populations. This heterogeneity exists within and across patient tumors, presenting a considerable challenge for therapeutic intervention. Classical genomic and molecular classification of GBM patients have identified four main genomic subtypes, proneural, neural, classical, and mesenchymal [5, 6], although additional molecular classifications and subtypes have also been determined [7, 8]. Despite this, it remains unclear whether the different genomic subtypes can predict patient response to anti-angiogenic treatments. Moreover, distinguishing GBM molecular subtypes has not yet let to improved patient outcomes [9].

Differential response to treatment has also been explored in the context of specific genetic mutations and structural aberrations. A recent pharmacological and genomic profiling of one hundred 100 patient-derived GBM cell cultures exposed to 1,544 drugs showed that the differential drug response could be linked to mutually exclusive aberrations in TP53 and CDKNK2A/B [10]. Mutant isocitrate dehydrogenase (mtIDH1) also appears to be predictive of treatment response. However, the roles of mutant IDH in cancer development and progression are possibly temporary or dynamic, making the timing of treatment with IDH inhibitorsof crucial importance [11].

Preclinical studies mainly focus on the inter-tumor heterogeneity across GBM patients, or a specific gene mutation which are mainly quantified in samples obtained from the resected tumor mass. However, GBM intratumoral molecular heterogeneity strongly limits the performance of therapies targeting specific mutations or molecular subtypes [9]. Conversely, the failure of current therapies to eliminate GBM subpopulations surrounding the edge of the tumor is considered to be the major factor contributing to the inevitable recurrence. The spatial heterogeneity within GBM tumors have been recently found to facilitate therapeutic resistance [12], where edge-derived cells show a higher capacity for infiltrative growth, while core cells demonstrate core lesions with greater therapy resistance. Understanding GBM intratumor heterogeneity is key to understanding treatment failure [13].

Intratumor heterogeneity for treatment stratification can be visualized during fluorescence-guided surgery. When 5-aminolevulinic acid (5-ALA) is administered orally prior to surgery, GBM cells glow fluorescent pink, which facilitates maximal resection [14]. However, the level of fluorescence emission varies across the tumor, which may be due to histological and genetic profiling of the tumor [15]. The heterogeneity of 5-ALA-induced fluorescence observed during surgery was associated with different cellular functions and a distinct mRNA expression profile [2]. Arguably, the reduction in 5-ALA fluorescence is suggestive of healthy tissue [16]. Regional variation of biomechanical properties within a tumor could influence therapeutic response and may be a factor that influences the fluorescence of 5-ALA acid-treated cells (see [17] for a review on the role of 5-ALA in addressing and characterizing cell heterogeneity in GBM research).

Recently, we demonstrated that tissue fluorescence and spatial heterogeneity are mirrored by physical heterogeneity among GBM primary cells [18]. Cells derived from weak and nonfluorescent tumor rim were smaller, adhered less well, and migrated quicker than cells derived from strongly fluorescent tumor mass. However, whether these properties can predict cellular response to treatments remains unclear. To investigate this hypothesis, we tested the response of tumor cells to two promising adhesion and metabolic inhibitors, GSK2256098/GTPL7939 (GSK), an ATP competitive reversible inhibitor of focal adhesion kinase (FAK) [19], and Gboxin, a metabolic inhibitor that selectively and irreversibly affects oxygen consumption in GBM cells [20]. GSK is currently in phase II clinical trials for solid cancers treatments such as adenocarcinoma, intracranial and recurrent meningioma mesothelioma and pancreatic cancer [21–26]. FAK is a non-receptor tyrosine kinase which is highly expressed in the nervous system [27] and is activated by cell-extracellular matrix adhesion [28]. FAK is known to play a crucial role in solid cancer progression [29–31]. FAK inhibitors that can selectively bind to the FAK ATP-binding domain are considered the most promising molecules to be translated and applied in clinical practice [32]. The ATP-competitive molecules bind to the FAK–kinase domain competing with ATP and, therefore, inhibiting FAK signal transduction activity and the activation of several FAK downstream pathways [33].

Gboxin is an inhibitor of F0F1 ATP synthase, which plays an important role in cancer metabolism including GBM [34]. A variety of promising F0F1 ATP synthase inhibitors have been reported [35–38], including Bedaquiline which is currently FDA approved [39]. Such inhibitors can offer targeted therapies, by targeting the oxidative phosphorylation pathway in the mitochondria which are dysfunctional in cancer cells [34]. Compared to normal cells, GBM cell have increased mitochondrial membrane potential. Gboxin spares normal cells and selectively targets GBM cells because of the loss of function of mitochondrial permeability transition pore in the cancer cells [20].

We demonstrate a substantial differential response to both treatments across different samples. Cells derived from highly fluorescent tumor core are significantly more resistant to Gboxin, and GSK, compared to cells derived from the weakly fluorescent tumor rim and nonfluorescent. Highly fluorescent cell proliferation ceased post-treatment in vitro when the ATP synthase inhibitor, Gboxin, was administered. While cells derived from non-fluorescent tumor margins exhibited higher potency for Gboxin, cell proliferation persisted post treatment. Since we already established that cells derived from the tumor core are more adherent and less migratory, our study suggests that the adhesive and migratory properties of cells may contribute to the variable sensitivity to both treatments across different regions of the tumor in individual patients, and between patients with GBM. Such heterogeneity may explain recently observed differential responses of patients to adhesion-blocking drugs.

## Materials and methods

### Sample collection

Tissue collection protocols complied with the UK Human Tissue Act 2004 (HTA license ref. 12,315) and have been approved by the local regional ethics committee (LREC ref. 04/Q0108/60). Tissue samples were derived as described in [18]. Briefly, tissues were derived from newly diagnosed GBM patients who underwent their first surgical resection at Addenbrooke’s, Cambridge University Hospitals. 5-ALA fluorescence was orally administered 4 h before induction of anesthesia at a dosage of 20 mg/kg. Three different regions within the tumor were biopsied. Six tissue samples from 2 different patients were taken from spatially separated sections using MRI stealth imaging. Navigated biopsy samples were collected from strongly fluorescent tumor cores, a weakly fluorescent tumor rim, and nonfluorescent tumor margins. The patient’s clinical information and their molecular biomarker status (IDH mutation and MGMT promoter methylation) can be found in Supplementary Table 1. We note that the cells obtained from both patients were IDH-wild type. There can be significant molecular differences between IDH-wild type and IDH mutant GBM cells [7, 40, 41]. However, about 90% of identified GBM patients have IDH-wild type primary tumors [8]. Hence, the results of this study are relevant to a vast majority of GBM cases.

### Derivation of GBM stem-like cells

Cell derivation and maintenance follows the protocols described in [42]. The tissue was mechanically minced and enzymatically dissociated before passing through a 40 µm cell strainer. Cells were seeded in serum-free medium (SFM; phenol red-free Neurobasal A) with 2 mM l-glutamine and 1% volume/volume (v/v) penicillin/streptomycin (PS) solution with 20 ng/mL human epidermal growth factor, 20 ng/mL zebrafish fibroblast growth factor (FGF-2), 1% v/v B27 SF supplement, and 1% N2 SF supplement. Cells were allowed to form primary aggregates. Spheroid aggregates were collected and plated onto Engelbreth-Holm-Swarm sarcoma extracellular matrix (ECM, Sigma)–coated flasks (ECM 1:50 dilution with HBSS) and allowed to form a primary monolayer. When the primary monolayer reached 80% confluency, cells were passaged to generate the subsequent monolayers by mechanically and enzymatically dissociating remaining aggregates. Cells were maintained at 37 °C and 5% CO2. Experiments were performed using passages 3–9, unless otherwise stated. Cell lines were screened regularly for mycoplasma.

### Cytotoxicity assays

Six thousand cells were cultured in 96 well plates (60 inner wells) coated with ECM at a concentration corresponding to a surface density of 6.67 μg/mL assuming complete adsorption. Cells were seeded in Triplicates. Following overnight incubation, cells were treated with increasing concentration of Gboxin (Cyman chemical) and GSK2256098 (Cayman Chemical) as indicated in the corresponding figure legends.

Gboxin viability assay was performed 40–42 h after treatment as per the protocol provided by the manufacturer for Cell Titer Glo® (Promega). This assay has previously been used to characterize the toxicity of Gboxin to glioblastoma cell lines [43, 44]. GSK viability assay was performed 18 h after treatment as per the protocol provided by the manufacturer for Thiazolyl blue tetrazolium bromide (Sigma). MTT and similar tetrazolium-based assays have previously been to study the effect of GSK on uterine cancer [45] and pancreatic ductal adenocarcinoma cells [46].

Blanks (outer wells, without cells) were included during the Cell Titer and MTT incubation for proper background subtraction. Data are expressed as a percentage of viability when compared with untreated cells (negative control—considered as 100% of viability). For the normalization of the data, treated cells were subtracted from cells treated with the corresponding dimethyl sulfoxide (DMSO) concentration present in each drug concentration. The 50% inhibitory concentration (IC50) for each drug was defined as the concentration producing 50% less viability compared to control wells.

### Polydimethylsiloxane (PDMS) substrates for studying cell survival and morphology post-treatment

Polydimethylsiloxane (PDMS) substrates were prepared as described in [18]. The use of PDMS allowed us to precisely control the stiffness of the cell environment. NuSil GEL-8100 (NuSil) was prepared in a 1:1 ratio of component A and component B and mixed well for 60 s: 1% (w/w) 10:1 (base/crosslinker w/w) Sylgard-184 (VWR) was added to the GEL-8100 and mixed well for 60 s. 80 mg per well of PDMS was added to 24-well culture plates (Corning Life Science). Coated vessels were baked at 65 °C for 13 h. This treatment gave a shear modulus value of G = 1.53 ± 0.12 kPa, which falls within the stiffness range experienced by infiltrating GBM cells [47]. The PDMS surface was coated with ECM at a surface density of 6.67 μg/mL, the same value used in the cytotoxicity assays. Ten thousand cells were cultured in 24 well plates. Following overnight incubation, cells were treated with Gboxin and GSK concentration which correspond to 50% ± 3% viability for the given cell line. Cells were seeded in triplicates and imaged every 10 min for 48 h. Time-lapse images were acquired with Zeiss Axio Observer Z1.

### Statistical analysis

Cell viability and proliferation data were collected from at least 3 independent biological experiments. The order of data collection was randomized; no blinding was performed, and no data were excluded from the analysis.

Representative results for biological replicates are shown for every figure except where specified otherwise in the figure legends. Data are presented as mean ± SEM as specified in the figure legend. Cell viability analysis was performed using the software GraphPad Prism 10.3.1.

For cell viability data, statistical significance with exact p value was determined using linear regression. First, we tested for differences between different patients and fluorescence groups independently. Afterward, we tested for differences simultaneously using an additive model (for patients and fluorescence intensity). To check whether sampling from multiple locations violated our standard linear model assumptions, we tested unexplained heterogeneity using mixed-effect regression. Including a random effect of all lines did not significantly improve model fit. This indicates that a mixed-effect model is not required. Statistical analyses and plotting were performed using the R statistical software package.

For cell proliferation data, we investigated the how drug treatment affected proportion of cells that underwent cell divisions. We used the Barnard’s test [48] on the pooled proliferation data from at least three biological replicates to test for statistically significant differences between control and treatment.

## Results

### Gboxin and GSK toxicity in GBM cells

**Fig. 1:**
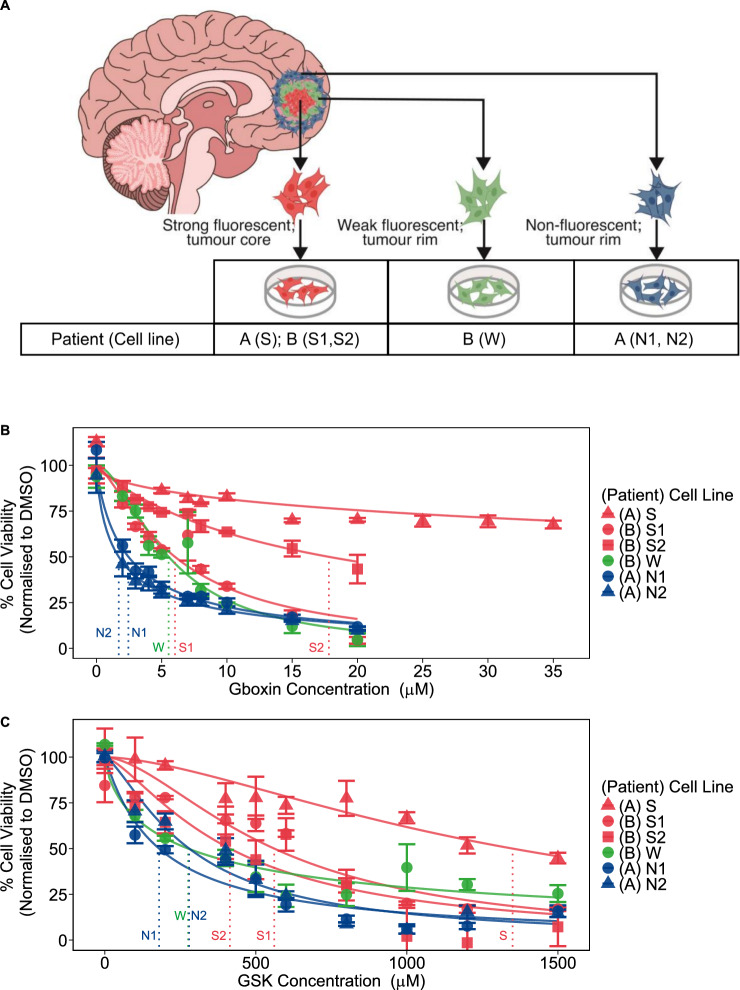
Differential treatment response of GBM cells to metabolic and adhesion inhibitors. **A** Collection of three strongly fluorescent tissue samples derived from the tumor mass (S from patient A, S1 and S2 from patient B, data shown in red on graphs), two nonfluorescent tumor margins (N1 and N2 from patient A, data in blue), and 1 weakly fluorescent tissue sample (W from patient B, data in green). The color code that represents different parts of the tumor is followed by the subsequent figures. **B** Cell viability (Cell Titer Glo assay) as a function of Gboxin concentration (in micromolar). Error bars represent mean ± SEM from three independent experiments. The response curves for each line is obtained from the mean of three biological replicates. The vertical lines mark the IC50 concentration of Gboxin for each cell line. **C** Cell viability (MTT assay) as a function of GSK concentration (in micromolar). Error bars represent mean ± SEM from three independent experiments. The response curves for each line is obtained from the mean of three biological replicates. The vertical lines mark the IC50 concentration of GSK for each cell line

To investigate GBM cellular response to adhesion and metabolic inhibitors, we tested different cell populations derived from different GBM tumors, as well as different regions from within the tumors (Fig. 1A). Cells were maintained in serum-free media following our established protocol [42] (see methods). We have previously confirmed the presence of glioma stem-like cells, cells expressed neural stem cell markers, nestin and vimentin [18] which are regarded as tumorigenic and associated with the heterogeneity in GBM [49]**.**

To understand the variability in cell toxicity observed between patient cell lines (Fig. 1B, Fig. 1C), we tested drug potency (IC50 values) across different patients. For each cell line and each drug, we fit a normalized inhibitor-response curve to every biological replicate, from which we obtained estimate mean and standard error for IC50 (Supplementary Fig. 1 and Supplementary Table 2).

To explain the variability in cell viability observed among patients’ cell lines, we first tested whether IC50 values cells from non-fluorescent tumor regions differed from those derived from strong and weak fluorescent regions using a simple linear regression (see Supplementary Table 3). Weakly and non-fluorescent cell lines W, N1, N2 exhibited higher GSK toxicity than strongly fluorescent cell lines S, S1, S2 (*P* < 0.05). However, no statistical difference in GSK toxicity was found between weakly and non-fluorescent cell lines. Moreover, when treated with Gboxin, no statistical significance in drug toxicity was found between the strongly, weakly or non-fluorescent cell lines.

We adjusted our model to account for the additive effects of both fluorescence intensity and patient identity (Methods, Supplementary Table 3). We found that strongly fluorescent cells derived from patient A were more resistant to both GSK and Gboxin than patient B (*P* < 0.001 and *P* < 0.001, respectively).

In addition, cells from non-fluorescent tumor had significantly higher toxicity to both drugs compared to cells derived from strongly (*P* < 0.001) and weakly fluorescent core samples (*P* < 0.001). The results demonstrate viability differences within each tumor and between tumors and show that this heterogeneity is related to 5-ALA fluorescence intensity.

### Cells derived from nonfluorescent tumor margins continue to proliferate post Gboxin treatment

To determine whether GSK and Gboxin will achieve the desired effect in vitro, we observed cell viability and cell division on a compliant PDMS substrate, for 24 h before administering both treatments, and for 18 h and 42 h after administering treatment for GSK and Gboxin respectively. Cells were cultured on a low modulus (1.5 kPa) PDMS, consistent with the stiffness of the environment that GBM infiltrate, which ranges from 0.1 to 10 kPa [47].

**Fig. 2:**
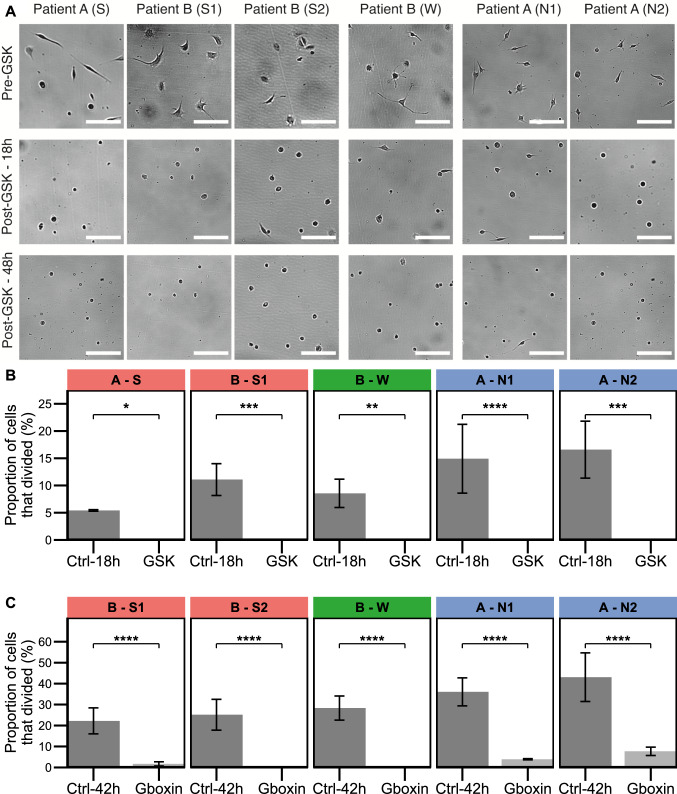
Effect of GSK and Gboxin treatments on GBM cell viability and cell division. Error bars, where appropriate, represent mean ± SEM at least three biological replicates. The number of asterisks indicate the magnitude of the *p*-value, P, obtained from Barnard’s test for statistical difference between control and treatment. (*P < 0.05, **P < 0.01, ***P < 0.0001, ****P < 0.000001). **A** Snapshots of cultured cells from different cell lines at before and after treatment with GSK (see Supplementary Fig. 2 for Gboxin). Scale bar: 100 µm. **B** Cell proliferation of the different cell lines following GSK treatment. **C** Cell proliferation of the different cell lines following Gboxin treatment

Strong, weak and non-fluorescent (S, S1, W, N1, N2) cell lines were treated with GSK at approximately the IC50 concentration (see Supplementary Table 4for exact concentrations). Prior to treatment, cell division was observed across all cell lines (*n* = 3) (Supplementary Video 1, Video 2 and Video 3). Around 65% of the cells treated with GSK lost their shape, detached, and become afloat within 18 h (*n* = 3), reaching 90% within 48 h (*n* = 3) (Fig. 2A). No cell division was observed across all cell lines for 8 h post GSK treatment (Supplementary Video 4, Video 5, Video 6).

We have then repeated the above experiment for Gboxin to investigate whether the cells would exhibit similar behavior. Interestingly, cell division and proliferation was observed for cells derived from non-fluorescent tumor margin (N1, N2) even when the treatment concentration was increased to IC50 + 30% and IC50 + 40% for the two lines, respectively. Strongly and weakly fluorescent cell lines were also treated with Gboxin at a concentration of IC50 ± 18% (see Supplementary Table 4for exact concentrations). Around 62% of the cells treated with Gboxin died/lost their shape, detached, and became afloat within 42 h (Supplementary Fig. 2).

Having qualitatively observed that the number of cell divisions was reduced post treatment, we then quantified the effect of treatment on proliferation. We determined the proportion of cells that can proliferate post treatment for each cell line and drug over the duration of the treatment, 18 h for GSK and 42 h for Gboxin (Fig. 2B, C).

The results show that cell division was significantly reduced post treatment for both drugs and all cell lines compared to pretreatment (Fig. 2B, C, Supplementary Videos). In the case of GSK, no cell division was observed for any cell line post treatment (Supplementary Videos 4, Video 5, Video 6). However, when Gboxin treatment was administered, cell division was significantly reduced in cells derived from strongly fluorescent tumor core and weakly fluorescent tumor rim; but a small number of divisions was observed by the cells from line (S1) (Fig. 2B, C, Supplementary Video 7, Video 8). In addition, cells derived from non-fluorescent tumor margins continued to divide post Gboxin treatment (Supplementary Video 9, Video 10), unlike the ones treated with GSK. Overall, the results suggest that both treatments lead to a significant reduction in observed cell proliferation for all cell lines, but cells derived from nonfluorescent tumor margins retain some proliferation after administering Gboxin treatment.

## Discussion

**Fig. 3:**
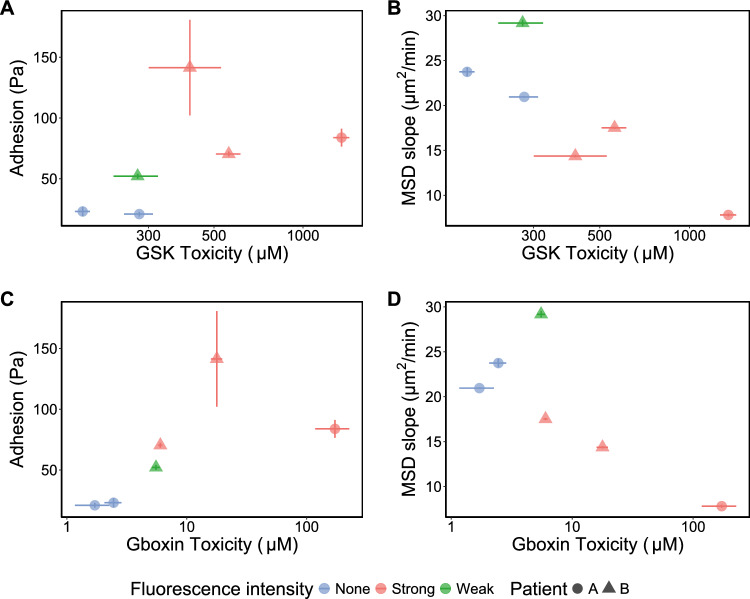
Relationship between GBM cell–matrix adhesion strength (characterized by the shear forces required to detach the cells from their substrate), cell migration (characterized by the slope of cell mean squared displacement (MSD)) [18] and drug potency (IC50 in micromolar) for different cell lines. **A** Relationship between GSK drug potency (IC50) and cell adhesion. Error bars represent SEM from at least 2 independent experiments. **B** Relationship between GSK drug potency (IC50) and cell migration. **C** Relationship between Gboxin drug potency (IC50) and cell adhesion. Error bars represent SEM from at least 2 independent experiments. D. Relationship between Gboxin drug potency (IC50) and cell migration

Our results reinforce that cells derived from highly fluorescent tumor core are significantly more resistant (an order of magnitude higher) to both GSK and Gboxin treatments, compared to cells derived from weakly fluorescent tumor rim and nonfluorescent tumor margins. Our earlier work demonstrated links between the fluorescence levels in the tissue and the cells’ mechanical behaviors, namely migration and adhesion [18]. Briefly, the adhesion strength was measured by the shear stress required to detach cells from the monolayer culture in a microfluidic setup. Cell migration was characterized by the mean squared displacement (MSD) of the cells, whose slope with time is a measure of cell motility; a higher MSD slope indicates more motile cells (see Supplementary Fig. 3for more details). Weak and nonfluorescent tumor rims were associated with smaller, less adherent and more migratory cells than the strongly fluorescent tumor mass. The relationship between cell adhesion and migration, obtained from the previous study, and drug toxicity from the current study is summarized below (Fig. 3). The distinct adhesive and migratory profiles of GBM cells suggest that patients’ differential response to adhesion inhibitors and anti-invasive molecular treatments may be linked to intrinsic differences in cell motility, adhesion, and traction forces.

As expected, the cell response to GSK treatment directly correlated with cell-ECM adhesion properties, which was also associated with the amount of 5-ALA fluorescence, and cell migration (Figs. 3A, B). In addition, GSK treatment prevented cell divisions in all the lines studied. These results are consistent with the previous studies which show the role of cell-ECM adhesion in cellular migration, invasion [50], and proliferation [51]. Highly adhesive, strongly fluorescent GBM stem cells were more resistant to GSK. This can be explained as FAK has a fundamental role in adhesion and is recruited to stabilize focal adhesions [52]. More adhesive cells are likely to have more FAK, as supported by the correlation between FAK expression and cell adhesion in glioblastoma cells [53]. Therefore, strongly fluorescent cells could be more resistant to FAK inhibitors and have increased survival due to a higher threshold for FAK-inhibition efficacy.

The results reveal that Gboxin potency inversely correlates with GBM cell migration (which was previously reported, Rezk et al. [18]). This was also associated with 5-ALA intensity and cell adhesion (Figs. 3C, D). The spatial distinction is in agreement with a recent study, by Bastola et al. [12], which showed that intratumor spatial heterogeneity facilitates therapeutic resistance where edge-derived cells show a higher capacity for infiltrative growth, and core cells demonstrated core lesions with greater therapy resistance in xenotransplantation, and in vitro radiation therapy [12].

A possible explanation lies in the Warburg effect, in which highly proliferative cells undergo a metabolic shift from oxidative phosphorylation, which is targeted by Gboxin, to aerobic glycolysis [54]. GBM intratumoral cell proliferation is strongly associated with the fluorescence intensity of samples and hence the spatial density and distribution of cells. Glioma cells with higher 5-ALA fluorescence were shown to be more proliferative than those with lower fluorescence [14]. This could explain the resistance exhibited by the strongly fluorescent tumor cells. In addition, this metabolic alteration is characterized by an increase in the ratio of oxidized and reduced forms of the carrier molecule Nicotinamide adenine dinucleotide (NAD + /NADH) [55, 56]. Yamashita et al. showed this ratio is higher at the core of GBM tumors than the periphery [57], implying a shift to aerobic glycolysis in the tumor core. Interestingly, while less proliferative cell lines (corresponding to non-fluorescent tumor margins) were more sensitive to Gboxin toxicity, their proliferation rate was disturbed less compared to other (strongly and weakly fluorescent) cell lines.

Preclinical studies of kinase inhibitors typically use lines from the resected tumor core/mass, which may not be representative of the distribution of the adhesion and motility properties of GBM edge-like cells. Indeed, GSK and Gboxin potency exhibited an identical pattern across cell lines. Cell viability assays investigating Gboxin potency on glioblastoma cell lines using Cell Titer Glo® assays have previously been published [43, 44], allowing us to compare our results (see Supplementary Table 5for IC50 values from these studies). While the duration of our treatment was shorter (42 h as opposed to 72 h), the values of IC50 were comparable to those previously published (10–38 μM [43, 44]), albeit only for strongly fluorescent lines (7–172 μM). The weakly and non-fluorescent cell lines had lower IC50 values (1.7–5.5 μM). This suggests that our results are comparable to previous studies investigating glioblastoma lines which are, potentially, more representative of strongly fluorescent tumor regions. The results also emphasize the importance of considering differential intertumoral response to treatment.

Our previous study showed that the adhesion proteins paxillin, vinculin, and FAK were expressed across all GBM cell lines, however, the colocalization of vinculin with F-actin was mainly evident at the edge of cells derived from strongly fluorescent lines [18]. The binding of vinculin to F-actin suggested spatial differences in focal adhesion assembly and enlargement within the tumor. This was also reflected by our findings from the cell detachment assays, where strongly fluorescent cells were found to be more adherent than weakly and non-fluorescent cells. While our study is limited by a small sample size, it shows that the spatial and intertumoral heterogeneity found in cell–matrix adhesion should be considered during therapeutic development/screening, particularly for therapeutics designed to target adhesion receptors or proteases. Future work should involve a more in-depth study with female patients, considering the established sexual dimorphism in genetic expression and treatment response [58], and an increased sample size to better characterise intertumoral heterogeneity.

We also demonstrate the importance of considering the heterogeneity of 5-ALA-induced fluorescence for drug screening [17]. The intratumor heterogeneity of 5-ALA-induced fluorescence during surgery and its genetic signature could be the key to understanding treatment failure and infiltration. Recent studies have revealed the molecular features of infiltrative 5-ALA-metabolizing cells which were associated with glioblastoma recurrence [59]. Our work demonstrates that highly fluorescent tumor core cells were significantly more resistant to Gboxin and GSK. While non-fluorescent, less adhesive cells [18] were less resistant, they continued to proliferate post Gboxin treatment. Future work is required to assess the gene signatures of these regions and how they correlate to the differential response to treatment [60, 61].

Therapeutic modalities could benefit from targeting the edge-located tumor-initiating cells from GBM patients [12]. Our study reveals that cells derived from both GBM tumor rim and tumor margins have a distinct response to treatment. It also showed that while drug potency was higher for cells derived from the edge-located tumor cells, they were still able to proliferate and grow in culture after Gboxin treatment. Our study reveals sampling is crucial and the cell adhesive and motility properties mechanics may play a role in drug sensitivity/screening.

## Supplementary Information

Below is the link to the electronic supplementary material.

Supplementary file1 (AVI 193092 KB)

Supplementary file2 (AVI 8716 KB)

Supplementary file3 (AVI 193092 KB)

Supplementary file4 (AVI 210360 KB)

Supplementary file5 (AVI 437984 KB)

Supplementary file6 (AVI 437984 KB)

Supplementary file7 (AVI 453683 KB)

Supplementary file8 (AVI 437984 KB)

Supplementary file9 (AVI 9915 KB)

Supplementary file10 (AVI 9751 KB)

## Data Availability

All numerical data reported in the paper as well as the scripts used to generate the plots are included as a zip file with the submission. The data will be placed in a public repository such as Zenodo before publication, and linked to the doi of the paper.
